# *tert*-Butyl 4-(3,4-dichloro­anilino)piperidine-1-carboxyl­ate

**DOI:** 10.1107/S1600536812051896

**Published:** 2013-01-09

**Authors:** Mehr-un-Nisa, Munawar Ali Munawar, Gabriel B. Hall, Sue A. Roberts, Victor J. Hruby

**Affiliations:** a Institute of Chemistry, University of The Punjab, Qaid-i-Azam Campus, Lahore 54590, Pakistan; b Department of Chemistry and Biochemistry, 1306 E University Boulevard, The University of Arizona, Tucson, AZ 85721, USA

## Abstract

In the title compound, C_16_H_22_Cl_2_N_2_O_2_, the substituted piperidine ring adopts a chair conformation with both substituents in equatorial positions. In the crystal, N—H⋯O and C—H⋯O hydrogen bonds connect mol­ecules into ribbons along the *a*-axis direction.

## Related literature

For the biological activity of piperazine derivatives, see: Hamed *et al.* (2012 ▶); Joergen *et al.* (1997 ▶); Peter *et al.* (2009 ▶). For the synthesis of the title compound, see: Vardanyan *et al.* (2009 ▶).
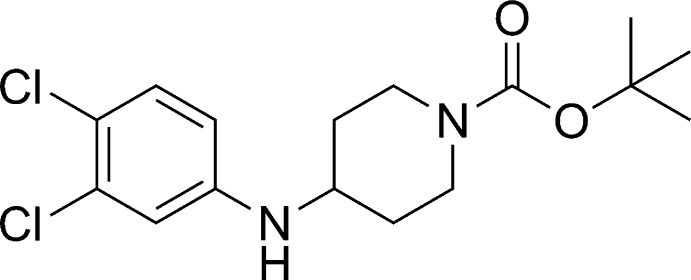


## Experimental

### 

#### Crystal data

C_16_H_22_Cl_2_N_2_O_2_
*M* *_r_* = 345.25
Orthorhombic, 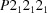

*a* = 9.7825 (6) Å
*b* = 10.6075 (6) Å
*c* = 16.8215 (10) Å
*V* = 1745.53 (18) Å^3^
*Z* = 4
Mo *K*α radiation
μ = 0.38 mm^−1^
*T* = 100 K
0.40 × 0.40 × 0.30 mm

#### Data collection

Bruker Kappa APEXII DUO CCD diffractometer
Absorption correction: multi-scan (*SADABS*; Bruker, 2009 ▶) *T*_min_ = 0.662, *T*_max_ = 0.749
34136 measured reflections
13197 independent reflections
11079 reflections with *I* > 2σ(*I*)
*R*_int_ = 0.024

#### Refinement

*R*[*F* ^2^ > 2σ(*F* ^2^)] = 0.034
*wR*(*F* ^2^) = 0.079
*S* = 1.00
13197 reflections
202 parameters
H-atom parameters constrained
Δρ_max_ = 0.44 e Å^−3^
Δρ_min_ = −0.19 e Å^−3^
Absolute structure: Flack (1983 ▶), 6110 Friedel pairs
Flack parameter: −0.01 (2)

### 

Data collection: *APEX2* (Bruker, 2009 ▶); cell refinement: *SAINT* (Bruker, 2009 ▶); data reduction: *SAINT*; program(s) used to solve structure: *SHELXS97* (Sheldrick, 2008 ▶); program(s) used to refine structure: *SHELXL97* (Sheldrick, 2008 ▶); molecular graphics: *OLEX2* (Dolomanov *et al.*, 2009 ▶); software used to prepare material for publication: *publCIF* (Westrip, 2010 ▶).

## Supplementary Material

Crystal structure: contains datablock(s) global, I. DOI: 10.1107/S1600536812051896/zl2529sup1.cif

Structure factors: contains datablock(s) I. DOI: 10.1107/S1600536812051896/zl2529Isup2.hkl

Supplementary material file. DOI: 10.1107/S1600536812051896/zl2529Isup3.cml

Additional supplementary materials:  crystallographic information; 3D view; checkCIF report

## Figures and Tables

**Table 1:** Hydrogen-bond geometry (Å, °)

*D*—H⋯*A*	*D*—H	H⋯*A*	*D*⋯*A*	*D*—H⋯*A*
N1—H1⋯O2^i^	0.88	2.12	2.9740 (8)	163
C3—H3⋯O2^i^	0.95	2.58	3.3486 (9)	138

Symmetry code: (i) 
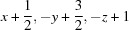
.

**Fig. 1.**
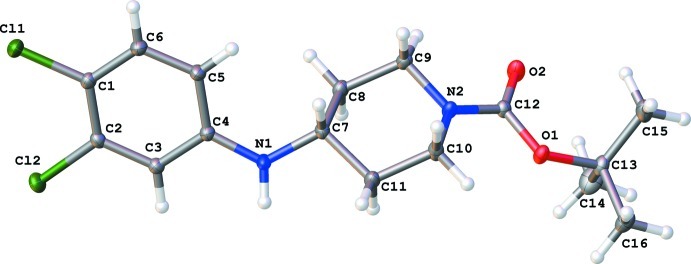
The molecular structure of the title compound. Anisotropically refined atoms are shown as 50% probability ellipsoids.

**Fig. 2.**
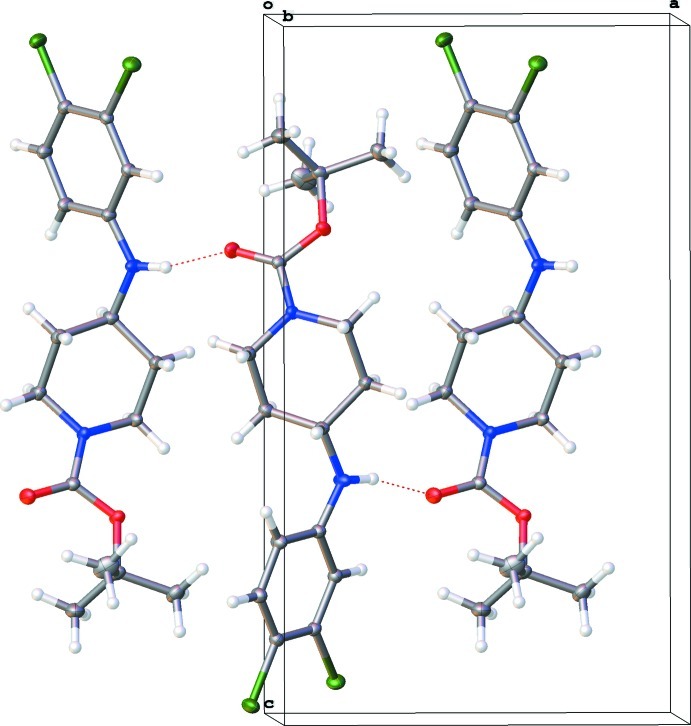
Hydrogen bonding interactions, shown as dashed lines. The molecules are connected into a chain running along the a direction in the crystal.

## supplementary crystallographic information

### Comment

Piperazine derivatives have been shown to inhibit re-uptake of the monoamines dopamine, noradrenaline and serotonin in synaptosomes (Joergen *et al.*, 1997), and their use for the treatment of protozoal infections, particularly malaria, has also been reported (Hamed *et al.*, 2012). Selective serotonin reuptake inhibitors (SSRI) provide efficacy in the treatment of numerous *CNS* disorders, including depression and panic disorders, and are usually observed to be effective, well tolerated and simply administered (Peter *et al.*, 2009)). During our search to find new synthetic novel multivalent ligands for the treatment of pain and depression, the title compound was synthesized as an intermediate. Compounds prepared from this intermediate are now under study for possible opioid and SSRIs activities.

The piperidine ring is in a chair conformation with both substituents in equatorial positions. An intermolecular hydrogen bond is present between N1—H1 and O2 with a donor-hydrogen-acceptor angle of 163.40° and a donor-aceptor distance of 2.9740 (8) Å. Hydrogen bonds connect molecules into ribbons extending in the crystallographic *a* direction. The hydrogen bonding graph set is C1,1(8)a.

### Experimental

*tert*-Butyl 4-((3,4-dichlorophenyl)amino)piperidine-1-carboxylate (1) was synthesized by modification of a reported method (Vardanyan *et al.*, 2009) by refluxing N-Boc-4-piperidone (5.0 g, 25.1 mmol) and 3,4-dichloroaniline (4.07 g, 25.1 mmol) in toluene (100 ml) with a catalytic amount of *p*-toluene sulphonic acid using a Dean and Stark apparatus for 4–5 h. The reaction was left to cool overnight. Toluene was evaporated under reduced pressure. The crude product was dissolved in diethyl ether, passed through a bed of neutral alumina, and the ether evaporated under reduced pressure. The residue was dissolved in CH_3_OH (50 ml) and NaBH_4_ (1.05 g, 27.6 mmol) was added slowly at room temperature. The reaction mixture was left to stir overnight. The reaction was quenched with aqueous NaHCO_3_ (5 ml). The solvent was removed under reduced pressure and the residue was dissolved in diethyl ether, dried over anhydrous MgSO_4_, filtered and evaporated under reduced pressure. The residue was recrystallized from CH_3_OH to obtain (1) as a white crystalline solid. Crystals appropriate for X-ray diffraction were grown from methanol by slow evaporation at room temperature. 8.22 g (95%) yield; m.p. 155–157 °C; MS (ESI): *m/z*: [*M*+H]^+^: 345; HRMS: Calcd for C_16_H_23_Cl_2_N_2_O_2_: 345.113; found: 345.1131.

### Refinement

All hydrogen atoms were visible in a difference Fourier map and were added at calculated positions. Bond distances are set to 0.95 Å for carbon-hydrogen bonds, and 0.88 Å for nitrogen-hydrogen bonds. Thermal parameters for hydrogen atoms were set to 1.2 times the isotropic equivalent thermal parameter of the atom to which the hydrogen atom is bonded.

### Crystal data

**Table:**

C_16_H_22_Cl_2_N_2_O_2_	*D*_x_ = 1.302 Mg m^−^^3^
*M**_r_* = 345.25	Mo *K*α radiation, λ = 0.71073 Å
Orthorhombic, *P*2_1_2_1_2_1_	Cell parameters from 9939 reflections
*a* = 9.7825 (6) Å	θ = 2.8–40.0°
*b* = 10.6075 (6) Å	µ = 0.38 mm^−^^1^
*c* = 16.8215 (10) Å	*T* = 100 K
*V* = 1745.53 (18) Å^3^	Rod, clear colourless
*Z* = 4	0.40 × 0.40 × 0.30 mm
*F*(000) = 728	

### Data collection

**Table:**

Bruker Kappa APEXII DUO CCD diffractometer	13197 independent reflections
Radiation source: fine-focus sealed tube	11079 reflections with *I* > 2σ(*I*)
Graphite monochromator	*R*_int_ = 0.024
Detector resolution: 8.3333 pixels mm^-1^	θ_max_ = 43.7°, θ_min_ = 2.3°
φ and ω scans	*h* = −19→17
Absorption correction: multi-scan (*SADABS*; Bruker, 2009)	*k* = −20→15
*T*_min_ = 0.662, *T*_max_ = 0.749	*l* = −32→22
34136 measured reflections	

### Refinement

**Table:**

Refinement on *F*^2^	Secondary atom site location: difference Fourier map
Least-squares matrix: full	Hydrogen site location: inferred from neighbouring sites
*R*[*F*^2^ > 2σ(*F*^2^)] = 0.034	H-atom parameters constrained
*wR*(*F*^2^) = 0.079	*w* = 1/[σ^2^(*F*_o_^2^) + (0.0361*P*)^2^ + 0.1129*P*] where *P* = (*F*_o_^2^ + 2*F*_c_^2^)/3
*S* = 1.00	(Δ/σ)_max_ = 0.002
13197 reflections	Δρ_max_ = 0.44 e Å^−^^3^
202 parameters	Δρ_min_ = −0.19 e Å^−^^3^
0 restraints	Absolute structure: Flack (1983), ???? Friedel pairs
Primary atom site location: structure-invariant direct methods	Flack parameter: −0.01 (2)

### Special details

**Table:**

Geometry. All e.s.d.'s (except the e.s.d. in the dihedral angle between two l.s. planes) are estimated using the full covariance matrix. The cell e.s.d.'s are taken into account individually in the estimation of e.s.d.'s in distances, angles and torsion angles; correlations between e.s.d.'s in cell parameters are only used when they are defined by crystal symmetry. An approximate (isotropic) treatment of cell e.s.d.'s is used for estimating e.s.d.'s involving l.s. planes.
Refinement. Refinement of *F*^2^ against ALL reflections. The weighted *R*-factor *wR* and goodness of fit *S* are based on *F*^2^, conventional *R*-factors *R* are based on *F*, with *F* set to zero for negative *F*^2^. The threshold expression of *F*^2^ > σ(*F*^2^) is used only for calculating *R*-factors(gt) *etc*. and is not relevant to the choice of reflections for refinement. *R*-factors based on *F*^2^ are statistically about twice as large as those based on *F*, and *R*- factors based on ALL data will be even larger.

### Fractional atomic coordinates and isotropic or equivalent isotropic displacement parameters (Å^2^)

**Table:**

	*x*	*y*	*z*	*U*_iso_*/*U*_eq_	
Cl1	−0.08164 (2)	0.97197 (2)	0.972015 (11)	0.02294 (4)	
Cl2	0.13863 (19)	0.75577 (18)	0.941766 (10)	0.01982 (3)	
O1	0.10534 (5)	0.83348 (5)	0.29230 (3)	0.01589 (9)	
O2	−0.11737 (5)	0.79311 (6)	0.32343 (3)	0.01988 (10)	
C14	0.06777 (12)	0.63102 (8)	0.22473 (6)	0.03006 (19)	
H14A	0.1276	0.595	0.2655	0.045*	
H14B	0.0822	0.5867	0.1743	0.045*	
H14C	−0.0278	0.6217	0.2412	0.045*	
C3	0.13364 (7)	0.82954 (6)	0.78917 (4)	0.01425 (10)	
H3	0.2006	0.7658	0.7815	0.017*	
C13	0.10078 (8)	0.77016 (7)	0.21416 (4)	0.01681 (11)	
C12	−0.00406 (7)	0.83543 (6)	0.34047 (4)	0.01344 (10)	
N2	0.02566 (6)	0.89021 (6)	0.41112 (4)	0.01450 (9)	
C10	0.15552 (7)	0.95171 (7)	0.43015 (4)	0.01648 (11)	
H10A	0.1433	1.0444	0.4296	0.02*	
H10B	0.2247	0.9295	0.3895	0.02*	
C11	0.20526 (7)	0.90986 (8)	0.51199 (4)	0.01733 (12)	
H11A	0.2881	0.9585	0.5263	0.021*	
H11B	0.2306	0.8196	0.5099	0.021*	
C7	0.09632 (7)	0.92909 (7)	0.57613 (4)	0.01480 (10)	
H7	0.0784	1.0215	0.5816	0.018*	
N1	0.14933 (7)	0.88223 (7)	0.65131 (4)	0.01898 (11)	
H1	0.2255	0.8381	0.6502	0.023*	
C4	0.08893 (7)	0.90197 (6)	0.72395 (4)	0.01433 (10)	
C5	−0.01262 (8)	0.99380 (7)	0.73731 (4)	0.01686 (12)	
H5	−0.0468	1.0417	0.6939	0.02*	
C6	−0.06310 (7)	1.01490 (7)	0.81332 (4)	0.01730 (11)	
H6	−0.1303	1.0783	0.8215	0.021*	
C1	−0.01660 (7)	0.94440 (7)	0.87772 (4)	0.01538 (11)	
C2	0.08092 (7)	0.85036 (6)	0.86435 (4)	0.01397 (10)	
C9	−0.08241 (7)	0.91113 (7)	0.46944 (4)	0.01573 (10)	
H9A	−0.1662	0.8659	0.4528	0.019*	
H9B	−0.1039	1.0022	0.4724	0.019*	
C8	−0.03699 (7)	0.86400 (7)	0.55099 (4)	0.01653 (11)	
H8A	−0.0228	0.7716	0.549	0.02*	
H8B	−0.1092	0.8818	0.5906	0.02*	
C16	0.24662 (8)	0.78782 (8)	0.18424 (5)	0.02256 (14)	
H16A	0.2688	0.8779	0.1832	0.034*	
H16B	0.2547	0.753	0.1305	0.034*	
H16C	0.3101	0.7439	0.2198	0.034*	
C15	0.00084 (9)	0.83714 (10)	0.15914 (5)	0.02569 (16)	
H15A	−0.0908	0.8352	0.1826	0.039*	
H15B	−0.0007	0.7944	0.1075	0.039*	
H15C	0.0297	0.9249	0.1519	0.039*	

### Atomic displacement parameters (Å^2^)

**Table:**

	*U*^11^	*U*^22^	*U*^33^	*U*^12^	*U*^13^	*U*^23^
Cl1	0.01965 (7)	0.03505 (10)	0.01412 (7)	0.00661 (7)	0.00311 (6)	0.00055 (6)
Cl2	0.02267 (7)	0.02154 (7)	0.01526 (6)	0.00232 (6)	−0.00074 (6)	0.00598 (6)
O1	0.0154 (2)	0.0189 (2)	0.01340 (19)	−0.00364 (16)	0.00210 (15)	−0.00379 (17)
O2	0.0162 (2)	0.0272 (3)	0.0163 (2)	−0.00867 (19)	−0.00053 (17)	−0.00255 (19)
C14	0.0419 (5)	0.0163 (3)	0.0320 (4)	−0.0073 (3)	0.0078 (4)	−0.0062 (3)
C3	0.0142 (2)	0.0149 (2)	0.0137 (2)	0.0010 (2)	−0.0004 (2)	0.0009 (2)
C13	0.0203 (3)	0.0166 (3)	0.0135 (2)	−0.0034 (2)	0.0022 (2)	−0.0035 (2)
C12	0.0144 (2)	0.0139 (2)	0.0120 (2)	−0.0024 (2)	−0.00017 (19)	0.00076 (19)
N2	0.0122 (2)	0.0189 (2)	0.0124 (2)	−0.00349 (18)	0.00084 (17)	−0.00202 (18)
C10	0.0145 (2)	0.0219 (3)	0.0131 (2)	−0.0061 (2)	−0.0004 (2)	0.0001 (2)
C11	0.0132 (2)	0.0253 (3)	0.0135 (3)	0.0000 (2)	−0.0002 (2)	−0.0005 (2)
C7	0.0151 (2)	0.0174 (3)	0.0119 (2)	0.0014 (2)	−0.00057 (19)	0.0005 (2)
N1	0.0201 (3)	0.0252 (3)	0.0116 (2)	0.0098 (2)	0.0005 (2)	0.0014 (2)
C4	0.0145 (2)	0.0160 (2)	0.0124 (2)	0.0019 (2)	0.0001 (2)	0.00067 (19)
C5	0.0173 (3)	0.0190 (3)	0.0142 (3)	0.0052 (2)	0.0003 (2)	0.0022 (2)
C6	0.0166 (3)	0.0199 (3)	0.0155 (3)	0.0044 (2)	0.0011 (2)	0.0010 (2)
C1	0.0140 (2)	0.0188 (3)	0.0133 (2)	0.0005 (2)	0.0013 (2)	0.0004 (2)
C2	0.0135 (2)	0.0150 (2)	0.0134 (2)	−0.0005 (2)	−0.0013 (2)	0.00250 (19)
C9	0.0132 (2)	0.0197 (3)	0.0143 (2)	−0.0003 (2)	0.0008 (2)	−0.0017 (2)
C8	0.0159 (3)	0.0197 (3)	0.0140 (3)	−0.0014 (2)	0.0024 (2)	0.0007 (2)
C16	0.0219 (3)	0.0250 (3)	0.0208 (3)	−0.0014 (3)	0.0062 (3)	−0.0042 (3)
C15	0.0246 (3)	0.0378 (4)	0.0147 (3)	0.0000 (3)	−0.0006 (3)	0.0007 (3)

### Geometric parameters (Å, º)

**Table:**

Cl1—C1	1.7338 (7)	C7—N1	1.4544 (9)
Cl2—C2	1.7382 (7)	C7—C8	1.5350 (10)
O1—C12	1.3425 (8)	C7—H7	1.0
O1—C13	1.4767 (8)	N1—C4	1.3734 (9)
O2—C12	1.2298 (8)	N1—H1	0.88
C14—C13	1.5213 (11)	C4—C5	1.4093 (10)
C14—H14A	0.98	C5—C6	1.3888 (10)
C14—H14B	0.98	C5—H5	0.95
C14—H14C	0.98	C6—C1	1.3928 (10)
C3—C2	1.3835 (10)	C6—H6	0.95
C3—C4	1.4090 (9)	C1—C2	1.3984 (10)
C3—H3	0.95	C9—C8	1.5261 (10)
C13—C15	1.5223 (12)	C9—H9A	0.99
C13—C16	1.5244 (11)	C9—H9B	0.99
C12—N2	1.3545 (9)	C8—H8A	0.99
N2—C9	1.4592 (9)	C8—H8B	0.99
N2—C10	1.4635 (9)	C16—H16A	0.98
C10—C11	1.5260 (10)	C16—H16B	0.98
C10—H10A	0.99	C16—H16C	0.98
C10—H10B	0.99	C15—H15A	0.98
C11—C7	1.5302 (10)	C15—H15B	0.98
C11—H11A	0.99	C15—H15C	0.98
C11—H11B	0.99		
			
C12—O1—C13	121.34 (5)	C4—N1—H1	117.7
C13—C14—H14A	109.5	C7—N1—H1	117.7
C13—C14—H14B	109.5	N1—C4—C3	118.43 (6)
H14A—C14—H14B	109.5	N1—C4—C5	123.40 (6)
C13—C14—H14C	109.5	C3—C4—C5	118.14 (6)
H14A—C14—H14C	109.5	C6—C5—C4	120.58 (6)
H14B—C14—H14C	109.5	C6—C5—H5	119.7
C2—C3—C4	120.59 (6)	C4—C5—H5	119.7
C2—C3—H3	119.7	C5—C6—C1	120.90 (7)
C4—C3—H3	119.7	C5—C6—H6	119.6
O1—C13—C15	110.38 (6)	C1—C6—H6	119.6
O1—C13—C16	102.11 (6)	C6—C1—C2	118.73 (6)
C15—C13—C16	110.06 (7)	C6—C1—Cl1	120.08 (5)
O1—C13—C14	110.10 (6)	C2—C1—Cl1	121.19 (5)
C15—C13—C14	112.80 (7)	C3—C2—C1	121.01 (6)
C16—C13—C14	110.89 (7)	C3—C2—Cl2	118.14 (5)
O2—C12—O1	124.91 (6)	C1—C2—Cl2	120.85 (5)
O2—C12—N2	123.67 (6)	N2—C9—C8	110.10 (6)
O1—C12—N2	111.42 (6)	N2—C9—H9A	109.6
C12—N2—C9	119.98 (6)	C8—C9—H9A	109.6
C12—N2—C10	124.71 (6)	N2—C9—H9B	109.6
C9—N2—C10	114.45 (6)	C8—C9—H9B	109.6
N2—C10—C11	110.14 (6)	H9A—C9—H9B	108.2
N2—C10—H10A	109.6	C9—C8—C7	110.34 (6)
C11—C10—H10A	109.6	C9—C8—H8A	109.6
N2—C10—H10B	109.6	C7—C8—H8A	109.6
C11—C10—H10B	109.6	C9—C8—H8B	109.6
H10A—C10—H10B	108.1	C7—C8—H8B	109.6
C10—C11—C7	112.04 (6)	H8A—C8—H8B	108.1
C10—C11—H11A	109.2	C13—C16—H16A	109.5
C7—C11—H11A	109.2	C13—C16—H16B	109.5
C10—C11—H11B	109.2	H16A—C16—H16B	109.5
C7—C11—H11B	109.2	C13—C16—H16C	109.5
H11A—C11—H11B	107.9	H16A—C16—H16C	109.5
N1—C7—C11	108.61 (6)	H16B—C16—H16C	109.5
N1—C7—C8	112.88 (6)	C13—C15—H15A	109.5
C11—C7—C8	109.72 (6)	C13—C15—H15B	109.5
N1—C7—H7	108.5	H15A—C15—H15B	109.5
C11—C7—H7	108.5	C13—C15—H15C	109.5
C8—C7—H7	108.5	H15A—C15—H15C	109.5
C4—N1—C7	124.62 (6)	H15B—C15—H15C	109.5
			
C12—O1—C13—C15	65.40 (8)	C2—C3—C4—N1	176.69 (7)
C12—O1—C13—C16	−177.61 (6)	C2—C3—C4—C5	−1.34 (10)
C12—O1—C13—C14	−59.79 (9)	N1—C4—C5—C6	−175.62 (7)
C13—O1—C12—O2	−4.42 (11)	C3—C4—C5—C6	2.31 (11)
C13—O1—C12—N2	175.50 (6)	C4—C5—C6—C1	−1.23 (12)
O2—C12—N2—C9	−5.18 (11)	C5—C6—C1—C2	−0.86 (11)
O1—C12—N2—C9	174.90 (6)	C5—C6—C1—Cl1	−179.73 (6)
O2—C12—N2—C10	−174.00 (7)	C4—C3—C2—C1	−0.72 (11)
O1—C12—N2—C10	6.08 (10)	C4—C3—C2—Cl2	178.95 (5)
C12—N2—C10—C11	−134.30 (7)	C6—C1—C2—C3	1.83 (11)
C9—N2—C10—C11	56.33 (8)	Cl1—C1—C2—C3	−179.31 (6)
N2—C10—C11—C7	−53.30 (8)	C6—C1—C2—Cl2	−177.83 (6)
C10—C11—C7—N1	177.63 (6)	Cl1—C1—C2—Cl2	1.03 (9)
C10—C11—C7—C8	53.82 (8)	C12—N2—C9—C8	131.49 (7)
C11—C7—N1—C4	169.17 (7)	C10—N2—C9—C8	−58.59 (8)
C8—C7—N1—C4	−68.92 (9)	N2—C9—C8—C7	56.87 (7)
C7—N1—C4—C3	166.47 (7)	N1—C7—C8—C9	−176.41 (6)
C7—N1—C4—C5	−15.61 (12)	C11—C7—C8—C9	−55.13 (8)

### Hydrogen-bond geometry (Å, º)

**Table:**

*D*—H···*A*	*D*—H	H···*A*	*D*···*A*	*D*—H···*A*
N1—H1···O2^i^	0.88	2.12	2.9740 (8)	163
C3—H3···O2^i^	0.95	2.58	3.3486 (9)	138

Symmetry code: (i) *x*+1/2, −*y*+3/2, −*z*+1.
